# The Continued Promise and Many Disappointments of Oncolytic Virotherapy in Gastrointestinal Malignancies

**DOI:** 10.3390/biomedicines5010010

**Published:** 2017-03-04

**Authors:** Daniel H. Ahn, Tanios Bekaii-Saab

**Affiliations:** Division of Hematology/Medical Oncology, Mayo Clinic, 5777 E. Mayo Blvd, Phoenix, AZ 85054, USA; ahn.daniel@mayo.edu

**Keywords:** oncolytic virotherapy, immunotherapy, targeted therapies

## Abstract

Oncolytic virotherapy represents a novel therapeutic strategy in the treatment of gastrointestinal malignancies. Oncolytic viruses, including genetically engineered and naturally occurring viruses, can selectively replicate in and induce tumor cell apoptosis without harming normal tissues, thus offering a promising tool in the armamentarium for cancer therapy. While this approach has garnered much interest over the past several decades, there has not been significant headway across various tumor types. The recent approval of talimogene laherparepvec, a second-generation oncolytic herpes simplex virus type-1, for the treatment of metastatic melanoma, confirms the therapeutic potential of oncolytic viral therapy. Herein, we will highlight and review the role of oncolytic viral therapy in gastrointestinal malignancies while discussing its limitations and potential alternative mechanisms to improve its treatment efficacy.

## 1. Oncolytic Virotherapy

Viral therapies represent one of many potential immunotherapeutic strategies in the treatment of gastrointestinal cancers. Historical evidence has shown tumor regression or remission of several advanced malignancies after the inoculation of naturally occurring viruses, including chicken pox, measles or hepatitis viral infections [1]. These early observations led to the preclinical investigation for viral therapies as a treatment for cancer. Viruses have the potential to preferentially infect and replicate in malignant cells while sparing normal healthy cells [2]. Cancer cell specific replication can be undertaken either by selecting a virus that is non-virulent to humans or by genetic modification of the virus genome.

The anti-cancer activities from oncolytic viruses are a result from the direct lysis of cancer cells by the virus, and by cytotoxicity to cancer and stromal cells by activated innate and tumor specific immune cells [3,4]. “Immunogenic” cell death of cancer and stromal cells results in the release of tumor-specific epitopes in conjunction with the damage-associated molecular pattern (DAMP) and oncolytic virus pathogen derived pathogen-associated molecular pattern (PAMP) molecules and inflammatory cytokines to elicit anti-tumor immunity [5,6,7]. Importantly, oncolytic viruses (OV) exhibit targeted anti-tumor activity against cancer stem cells (CSCs), which are tumorigenic and responsible for tumor invasion and treatment resistance [8].

The ability of oncolytic viral therapy to induce tumor cell apoptosis and stimulate an anti-tumor immune response has resulted in its interest as a potential treatment across various tumor types, including in the treatment of gastrointestinal malignancies, and will be reviewed in detail below (Table 1).

## 2. Reovirus

Respiratory enteric orphan virus (Reovirus) is a double-stranded RNA virus that occurs naturally in humans and can infect the gastrointestinal system and respiratory tract. Its infection is considered minor in humans, typically causing symptoms in early childhood and adolescent adults, where Reovirus exhibits cytopathic effects and oncolytic potential in cancer cells. Reovirus is dependent upon the cellular activity of *RAS*; thus activated *RAS* signaling contributes to its tumor-specific viral replication and oncolytic properties [14,15,16,17].

Early preclinical studies with Reovirus demonstrated its ability to infect, replicate and induce cancer cell specific apoptosis with no activity in normal tissue across several gastrointestinal malignancies [18,19,20]. Reolysin (pelareorep) is a propriety formulation of the naturally occurring Reovirus Serotype3-Dearing strain, a live replication-competent Reovirus that has demonstrated cytotoxic effects on cancer cells that harbored mutations in the *RAS* signaling pathway with a tolerable safety profile [17,21,22,23,24]. Further, when administered concomitantly with chemotherapy or radiotherapy, early preclinical anti-tumor activity suggested a potential benefit from a combined modality approach with Reolysin [25,26].

Given the ubiquitous nature of *RAS* mutations in colorectal and pancreatic cancer [27,28], Reolysin has been of interest as a novel therapeutic agent in the treatment of these diseases. In preclinical studies, Reolysin induced endoplasmic reticulum stress-mediated oncolytic activity in *RAS* mutated pancreatic cancer cell lines [29,30]. In early phase safety trials, the intratumoral injection of Reolysin into pancreatic cancer tumors resulted in tumor regression. In addition to tumor growth suppression, immunohistochemical studies confirmed the presence of Reovirus in each of the responding tumors.

A phase I dose escalation study was conducted in patients with treatment refractory *KRAS* mutant colorectal cancer that received the combination of Reolysin with FOLFIRI (5-Fluorouracil (5-FU) and Irinotecan) [31]. The combination was safe and well tolerated with encouraging preliminary activity, where 55% of (10 of 18) patients experienced disease control (stable disease or partial response). Based on these findings, IND210, a randomized phase II study, investigated the combination of FOLFOX (5-FU and Oxaliplatin) and Bevacizumab with or without Reolysin (clinicaltrials.gov, NCT01622543). The preliminary results did not demonstrate a significant clinical benefit with the addition of Reolysin to standard chemotherapy in colorectal cancer.

In pancreatic adenocarcinoma, several clinical trials with Reolysin added to chemotherapy were conducted. Noonan et al. reported the results of a randomized phase II trial where 73 treatment-naïve patients with metastatic pancreatic adenocarcinoma were randomized to receive carboplatin/paclitaxel alone or in combination with Reolysin [11]. While this agent was well tolerated with minimal treatment-related adverse effects, it failed to show an improvement in clinical outcomes, including in patients whose tumors exhibited *KRAS* mutations. Interestingly, increased levels in immunomodulating biomarkers (including Interleukin (IL)-6, Vascular Endothelial Growth Factor (VEGF), and regulatory T cells) were seen in patients that received Reolysin. These results are consistent with preclinical studies that suggest Reolysin may promote and enhance immune suppression in a pre-existing immunosuppressive environment that is seen in pancreatic adenocarcinoma [32]. A second single arm phase II study evaluated the combination of Reolysin with gemcitabine in treatment-naïve patients with advanced pancreatic adenocarcinoma. Of the 33 patients that were enrolled, a median progression free survival of 4 months and overall survival of 10.2 months were observed. Only one patient had a partial response, 23 had stable disease and five patients experienced disease progression. These findings did not show any significant improvement in patient outcomes when compared to historical standard therapies in advanced pancreatic adenoarcinoma [33,34].

Overall, the lack of demonstrable clinical activity with Reolysin across a spectrum of gastrointestinal malignancies was very disappointing, and this agent is a very unlikely candidate for further development in this space.

## 3. Adenovirus

Adenoviruses (members of the family Adenoviridae) are non-enveloped, linear double-stranded DNA viruses that have shown oncolytic activity across a large spectrum of tumor types. It was also the first oncolytic virus to be evaluated in a clinical trial for the treatment of pancreatic adenocarcinoma. ONYX-015 is an E1B 55-kDA region deleted adenovirus that preferentially replicates in cancer cells with *p53* alterations, but not in adjacent normal cells. Its first indication as a therapeutic option came in 2005 after the approval of H101, an oncolytic adenovirus similar to ONYX-015 for the treatment of nasopharyngeal cancer or esophageal cancer in China [35]. Based on early preclinical studies that confirmed viral adenovirus replication in abnormal cells, a phase I study evaluating intratumoral administration of ONXY-015 was conducted in patients with advanced pancreatic adenocarcinoma. While no objective responses were seen in the 23 enrolled patients, 11 patients experienced stable disease for at least 12 weeks [35]. The treatment was well tolerated with minimal adverse effects. Subsequently, a phase I/II study was conducted in patients with unresectable pancreatic adenocarcinoma, where ONYX-015 was administered intratumorally under endoscopic ultrasound-guidance in combination with intravenous gemcitabine chemotherapy [10]. Of the 21 patients enrolled in the study, two patients experienced a partial response. Treatment-related adverse effects were mild, with primarily injection-related complications (such as local infection and duodenal perforation) were observed. Although deemed safe and tolerable, there was no significant activity observed from these two studies.

In attempts to further enhance anti-tumor activity with the clinical application of adenovirus, genetic modifications have been employed. One strategy includes designing viral therapies that target tissue-specific promoters to control viral gene selective expression in tumor specific cells. For example, GD55 was developed as a genetically modified oncolytic adenovirus designed for the treatment of hepatocellular carcinoma (HCC) that targets GOLPH2, a Golgi membrane glycoprotein GP73 that is specific for this disease [36]. In preclinical studies, GOLPH2-regulated GD55 conferred higher adenovirus replication and infectivity with significant tumor growth suppressing activity with no effect on surrounding normal tissue [37]. Recent preclinical studies investigating GD55 have reproduced similar findings with induced-apoptosis inhibited dissemination of liver cancer stem cell-like cells [38], thus reaffirming GD55 as a potential therapeutic option in HCC. Currently, the utility of modified adenovirus as a potential viable treatment option in gastrointestinal malignancies remains in its early preliminary stage where further investigation is needed prior to application in the clinic.

## 4. Herpes Simplex Virus (HSV)

Oncolytic HSV vectors have been investigated widely in various malignancies, including gastrointestinal malignancies, despite a primary interest in their therapeutic role in neurological malignancies given their neurotropic nature. The therapeutic potential has been validated with the recent Food and Drug Adminstration approval of Talimogene laherparepvec (T-Vec, tradenamed Imlygic, formerly Oncovex^GM−CSF^), for the treatment of advanced, unresectable melanoma.

T-Vec is a genetically modified double mutated HSV-1 oncolytic virus with deletions in the γ34.5 and α47 genes and the insertion of human granulocyte-macrophage colony-stimulating factor (GM-CSF) gene into the deleted γ34.5 loci. The deletion in the γ34.5 genes allows for selective replication within cancer cells while leaving normal tissue unharmed [39,40,41]. OPTiM, a randomized open-label phase III trial, evaluated the role of T-Vec in unresectable stage IIB-IV melanoma where 436 patients were randomized in a 2:1 fashion to receive either intratumoral T-Vec or subcutaneous GM-CSF, where the primary endpoint was a durable response rate (partial or complete response continuously for ≥6 months starting within 12 months). Patients who received T-Vec experienced a durable response rate of 16.3% (response >6 months) of which 29.1% had a complete response [42]. Treatment was well tolerated where the only grade 3 or 4 treatment related adverse event occurring in greater than 2% of patients was cellulitis (*n* = 6 in the T-VEC arm; *n* = 1 with GM-CSF). This study was the first to demonstrate that local intratumoral injection provides local tumor growth suppression and through a presumed abscopal systemic effect. A more recent phase I study with intratumoral Oncovex GM-CSF injection in unresectable pancreatic cancer (clinicaltrials.gov, NCT00402025) has been completed and will provide further insight regarding a clinical role for this agent. Additionally, an ongoing phase I trial is examining the abscopal effect of radiation therapy with T-Vec in other solid tumor malignancies (including gastrointestinal cancers) by examining the combination of T-Vec with or without radiotherapy (clinicaltrials.gov, NCT02819843). Several early phase studies have been completed in gastrointestinal malignancies evaluating the role of HSV-1 as a potential therapeutic option. In Japan, a phase I study was conducted where patients with unresectable pancreatic adenocarcinoma received intratumoral injections with HF-10, an unadulterated naturally occurring oncolytic HSV. No treatment related adverse effects were observed and four patients experienced a clinical benefit (partial response or prolonged stable disease) [13]. To further enhance anti-tumor activity, G47 Δ, a triple mutated third-generation oncolytic HSV-1 was developed with the goal of augmenting viral replication as demonstrated in a number of preclinical studies in a number of solid tumor models including hepatocellular carcinoma [43]. A number of phase I-II studies have now been completed in glioblastoma. However, currently, no clinical trials are ongoing in gastrointestinal malignancies.

## 5. Vesicular Stomatitis Virus (VSV)

VSV is a RNA virus that is non-cell cycle dependent, resulting in rapid uptake by cells. The Indiana strain of VSV and its recombinant derivatives have shown promising oncolytic activity in various malignancies [44,45]. It has a rapid cell cycle that is evidenced by 24-h post-infection cell apoptosis. In pancreatic cancer cell lines, VSV showed superior oncolytic activity in comparison to RSV and sendai virus. Resistant cell lines demonstrated that viral replication was dependent on type 1 interferon (IFN) activity, where highly viral replication and infectivity was seen in pancreatic cancer cell lines that lacked an intact IFN response [5]. Additionally, in the same resistant cell lines, a high level expression of anti-viral IFN-stimulated genes, *MxA* and *OAS* and aberrant activation of the *JAK*/*STAT* signaling pathway were observed; these findings may potentially limit the ability for the virus to adequately infect cancer cells [46,47].

VSV, however, is sensitive to the anti-viral effects of interferon (IFN)-α and β produced by infected cells, which limit its potential replication and propagation to neighboring cells. Thus, genetically modified oncolytic VSVs that express human interferon-β (VSV-hIFNβ) are in development in an attempt to induce high levels of IFN and protect the neighboring normal cells while retaining its cytotoxicity effect against tumor cells [48].

In early preclinical studies conducted in hepatocellular carcinoma and colorectal tumor xenografts in mice models, IFN-inducing VSVs have shown significant oncolytic activity with extensive tumor necrosis and prolongation of survival [49,50]. Based on this promising anti-tumor activity, a phase I dose escalation clinical trial (clinicaltrials.gov, NCT01628640) was developed and is ongoing. In this study, patients with HCC receive a single intratumoral injection with recombinant VSV-hIFNβ. The preclinical toxicology and pharmacology studies should provide further insight into the safety, tolerability and preliminary anti-tumor activity of VSV-hIFNβ in patients with hepatocellular carcinoma.

Current work to modulate IFN signaling through genetic modification or in combination with small molecule inhibitors of the cellular signaling pathway may enhance the oncolytic activity of VSV. A phase I study (clinicaltrials.gov, NCT02923466) is examining the safety and tolerability from intratumoral administration of genetically modified VSV that expresses IFN in treatment refractory solid tumors.

## 6. Vaccinia Virus

Vaccinia virus (VV) is a replication competent virus and is a member of the poxvirus family that has demonstrated promising anti-tumor activity, including in a number of gastrointestinal malignancies. It is highly immunogenic and produces a strong cytotoxic T cell and innate immune response [51]. In colon cancer, oncolytic VV has demonstrated interesting activity in preclinical studies and has shown synergistic activity with chemotherapy with an ability to overcome treatment resistance and enhance suppression of colon cancer stem cells, which are intrinsically resistant to chemotherapeutic agents [52,53].

Vaccinia (GLV-1h68) has also shown promising activity as a single agent or in combination with gemcitabine chemotherapy in pancreatic adenocarcinoma, where vaccinia has shown the ability to infect, replicate and induce oncolysis in pancreatic cancer cell lines [54]. GLV-1h51, a similar genetically modified vaccinia virus, has been tested across several cancer models. In one study, pancreatic cancer cell lines were found to be most sensitive to viral infection, resulting in decreased mean tumor volume [55]. As such, an ongoing phase I study in solid tumors, including pancreatic adenocarcinoma, (clinicaltrials.gov, NCT02432963) is investigating the combination of a modified vaccinia virus Ankara vaccine that expresses p53 with pembrolizumab, an anti-PD1 inhibitor, in treatment refractory patients.

Another oncolytic VV under clinical investigation is JX-594, a genetically modified VV strain with the deletion of the thymidine kinase gene that enhances preferential replication and lysis of proliferating cancer cells [56]. Its ability to self-amplify in tumors was confirmed in early phase studies where JX-594 was well tolerated when both intratumoral injection and intravenous infusions were applied [57,58,59]. While safety and tolerability was performed in early phase studies in patients with diverse cancer types, it was notable that patients who experienced the longest survival benefit were those with the highest complement-dependent cytotoxicity (CDC), determined by percentage cell viability (>50% cell killing) and ELIPSOT analysis to detect T cells producing interferon-γ at baseline and after JX-594 treatment [12]. These findings were observed initially in hepatocellular carcinoma, where a response rate of 15% and median overall survival of up to 14.1 months were recently reported in a dose escalation study [12]. Based on this observed clinical activity, PHOCUS, a randomized phase III trial, is being conducted with the combination of JX-594 plus sorafenib versus sorafenib in patients with hepatocellular carcinoma (clinicaltrials.gov, NCT02562755).

## 7. Challenges with the Clinical Development of Oncolytic Virotherapy

The rationale for oncolytic virotherapy as a viable therapeutic option for gastrointestinal malignancies remains exciting, however many limitations must be overcome. An increased understanding of the regulation of the anti-tumor response is needed to allow for the development of more rational clinical treatment strategies in order to improve on its efficacy.

A significant barrier for clinical application is the immunologic tolerance towards cancer specific antigens. Most tumor associated antigens are self-antigens and thus are weakly immunogenic. Additionally, the immunosuppressive tumor microenvironment suppresses the activity of tumor infiltrating lymphocytes, thus blunting anti-tumor immunogenic activity [60]. Therefore, effective anti-tumor response requires lessening in immunologic tolerance while increasing the tumor specific antigen exposure with enhanced tumor cell lysis. Immunotherapeutic approaches—notably agents that target negative immunologic regulatory molecules on activated T cells, such as programmed death-1 and its binding ligand, programmed death ligand 1 (PD-L1)—have shown promise as a therapeutic agent in various solid tumor malignancies [61]. The combination of an oncolytic virus with PD-1 or PD-L1 checkpoint inhibitors can potentially enhance and produce a synergistic anti-tumor immune response [62].

The mode of administration of the specific oncolytic virus also influences the effectiveness of its anti-tumor activity. While intravenous administration may be more practical, it is limited by hepatic and splenic sequestration or pre-existing serum anti-viral antibodies, which may result in insufficient viral particle delivery. Studies evaluating strategies to improve oncolytic virus (OV) distribution include modification of the viral coat proteins to evade antibody recognition and the utilization of mesenchymal stem cell carriers to increase the target delivery of the OV. Physical barriers can also influence the effectiveness of virotherapy. The stroma creates a complex microenvironment that not only serves as a physical barrier to prevent effective penetration of oncolytic viruses, but also provides an immunosuppressive environment that blunts the innate anti-tumor immune response [63,64,65,66,67,68,69,70]. Strategies deploying biologic therapies (such as interleukin-2, or anti-VEGF agents) can modulate the microenvironment by depleting immunosuppressive regulatory T cells that can potentiate the effect from the OV [71,72].

## 8. Conclusions and Future Directions

Despite the many promising advances, including recent FDA approval of T-vec in metastatic melanoma, the field of oncolytic virotherapy remains largely investigational in gastrointestinal malignancies. Early preclinical activity has not translated to meaningful clinical activity in larger randomized studies. An increased understanding in mechanisms of immunologic tolerance and treatment resistance has provided a stronger rationale that has spurred further clinical investigation. Through applications of innovative genetic modifications and combinatory strategies with novel immunotherapeutic agents including checkpoint inhibitors (e.g., PD-1 inhibitors or anti CTLA-4 antibodies) or biologic therapies (including anti-angiogenic therapies or Inodleamine 2,3-dioxygenase (IDO) inhibitors), oncolytic virotherapy will hopefully find a future role in the treatment of gastrointestinal cancers.

## Figures and Tables

**Table 1:** Summary of key completed phase I/II clinical trials investigating oncolytic viruses in gastrointestinal malignancies.

Study	Virus	Phase	Primary Endpoint	Median PFS *	Median OS *	Comments	Primary Tumor Site and Histology	Ref/NCT
Mulvihill et al.	Adenovirus	I	Safety, tolerance	N/A	N/A	Eleven of 23 pts with SD	Pancreatic Adeno Ca	[9]
Hetcht et al.	Adenovirus	I/II	Safety, tolerance	N/A	N/A	Administered IT with IV gemcitabine. Of 21 pts, two pts with PR, four with SD	Pancreatic Adeno Ca	[10]
Noonan et al.	Reovirus	II	PFS	4.9	7.31	IV with carboplatin and paclitaxel for trmt naive pts	Pancreatic Adeno Ca	[11]
	Control (carboplatin and paclitaxel)			5.2				
Mahalingam et al.	Reovirus	II	PFS	4	10.2	trmt naive pts; single arm	Pancreatic Adeno Ca	NCT00998322
Ocean et al.	Reovirus	I	Safety, tolerance	7.4	N/A	Ten of 18 pts experienced SD or better; in combination with FOLFIRI	Colon Adeno Ca	NCT01274624
Heo et al.	Vaccinia (JX-594)	II	Determine optimal dosing (low vs high)		14.1 (high dose) vs. 6.7 (low dose)		Liver (Hepatocellular carcinoma)	[12]
Nakao et al.	Herpes	I	Safety, tolerance	N/A	N/A	Of six pts, three pts with SD, one pt with PR	Pancreatic Adeno Ca	[13]
Senzer et al.	Tvec	I	Safety, tolerance	N/A	N/A	Intratumoral injection	Pancreatic Adeno Ca	00402025

PFS—progression free survival; OS—overall survival; SD—stable disease; PR—partial response; IV—intravenous; trmt—treatment; Ca—cancer; N/A—not applicable; Ref—references; IT—intratumorally; pts—patients. *—In months; NCT—National Clinical Trial.
